# Eyes absent gene (EYA1) is a pathogenic driver and a therapeutic target for melanoma

**DOI:** 10.18632/oncotarget.21352

**Published:** 2017-09-27

**Authors:** Joshua Jiawei Zhou, Yuanshen Huang, Xue Zhang, Yabin Cheng, Liren Tang, Xiaodong Ma

**Affiliations:** ^1^ Department of Anesthesiology, Pharmacology, and Therapeutics, Faculty of Medicine, University of British Columbia, Vancouver, BC, Canada; ^2^ Welichem Biotech Inc., Burnaby, BC, Canada; ^3^ Department of Dermatology and Skin Science, University of British Columbia, Vancouver, BC, Canada; ^4^ School of Pharmaceutical Sciences, Xiamen University, Xiamen, Fujian, China; ^5^ College of Pharmacology, Dalian Medical University, Dalian, Liaoning Province, China

**Keywords:** melanoma, biomarker, EYA1, chromatin remodeling, benzbromarone

## Abstract

EYA1 is a DNA repair enzyme that is induced after DNA damage and is upregulated in melanoma. However, its role in pathogenesis and therapeutic targeting of melanoma is unknown. Our objectives are (1) to study the relationship between EYA1 expression levels and melanoma patients’ clinical pathologic parameters including survival; (2) to investigate its impact on cultured melanoma cells *in vitro*; and (3) to evaluate EYA1 inhibitors’ potential as a treatment of melanoma.

Melanoma tissue microarrays were used to assess EYA1 protein expression in 326 melanoma tissues, and to correlate the expression with patients’ clinical pathological parameters. In addition, retroviral ShRNA vectors were used to silence expression of EYA1 in A375 melanoma cells, and the resultant cells examined for changes in growth, DNA synthesis, and tumor formation *in vitro*. Lastly, melanoma cells were treated with benzbromarone with or without the BRAF inhibitor vemurafenib.

Our results showed that EYA1 protein is low in benign nevi, but is significantly up-regulated in melanoma *in situ*, and remains high in invasive and metastatic melanoma. In addition, silencing of EYA1 gene expression resulted in decreased proliferation and colony formation. These were associated with decreased cyclin D1 and increased phosphorylated histone protein γH2AX. Finally, treatment with benzbromarone, a specific inhibitor of EYA1, caused significant inhibition of melanoma cell proliferation, and increased sensitivity to the BRAF inhibitor vemurafenib.

In conclusion, EYA1 gene is a pathogenic driver in melanoma pathogenesis. Targeting EYA1 may be a valuable strategy for treatment of melanoma.

## INTRODUCTION

The incidence and mortality of melanoma continue to increase in the world, resulting in more than 10,000 deaths in the US in 2016 [1]. Although significant advances have been made in the development of new therapies for melanoma, especially in targeted and immunotherapy such as BRAF/MEK inhibitors and checkpoint inhibitor blockade therapies [2], more than half of patients with advanced melanoma succumb to the disease. Therefore, new therapeutic approaches are still needed, necessitating the need to identify additional therapeutic targets of melanoma by improving understanding of melanoma pathogenesis.

A large body of evidence support that cutaneous melanoma pathogenesis follows a multi-stepped progression, each step marked by functional genetic mutations and distinct patterns of gene expression [3, 4]. Previous research from our group [5–13] and others [3, 14–20] have revealed numerous genes significantly upregulated in melanoma biopsies compared with benign nevi, and many of these have been shown to contribute to melanoma pathogenesis in *in vitro* and *in vivo* experimental systems [21, 22]. The genes implicated in melanoma development have been demonstrated to be involved in regulation of cell cycle progression, extracellular matrix remodeling, migration, apoptosis resistance, and many other pathways. However, many more genes enriched in melanomas have remained uncharacterized. One of these genes is EYA1, which is found to be over expressed in melanoma transcriptome analysis [23].

EYA1, or the “eyes absent” gene, was originally discovered as a developmentally essential gene in *Drosophila*, where its knockout resulted in absence or malformation of eyes, thus the name of “Eyes Absent” [24]. In humans, loss of function mutations results in Branchio-oto-renal Syndrome [25], which is characterized by developmental defects in the ear and kidney.

EYA1 gene encodes a phosphatase that plays a critical role in DNA repair [26]. Damages in chromatin causes phosphorylation of histones that result in apoptosis when the DNA damages are not repaired [27]. However, when EYA1 is expressed, it causes dephosphorylation of these histones, promoting DNA repair and cell proliferation [27]. Specifically, EYA1 removes the phosphate group of Y142-p on γH2AX [27]. Fluorescence studies have shown that the UV or IR-induced double stranded DNA breakage causes upregulation of EYA1 levels [27].

EYA1 has been shown to play a critical role in other cancers [28, 29]. In breast cancer EYA1 levels are increased, which promotes proliferation by activating cyclin D1. Further, this action is directly dependent upon its histone phosphatase activity [27]. However, its role in melanoma is currently unknown.

In this study, we examined the expression of EYA1 in a spectrum of melanocytic and non-melanocytic neoplasms of the skin, investigated its correlation with clinical-pathological parameters of melanoma, characterized its functional role in melanoma cells, and evaluated the therapeutic potential of EYA1 inhibitor benzbromarone as a therapeutic agent for melanoma cells. Our results revealed that EYA1 expression is associated with malignant transformation of melanoma cells but not that of keratinocyte malignancies. Further, EYA1 gene silencing and inhibition significantly decreased proliferation of melanoma cells.

## RESULTS

### EYA1 is increased in melanoma tissues and melanoma cells

**Figure 1:**
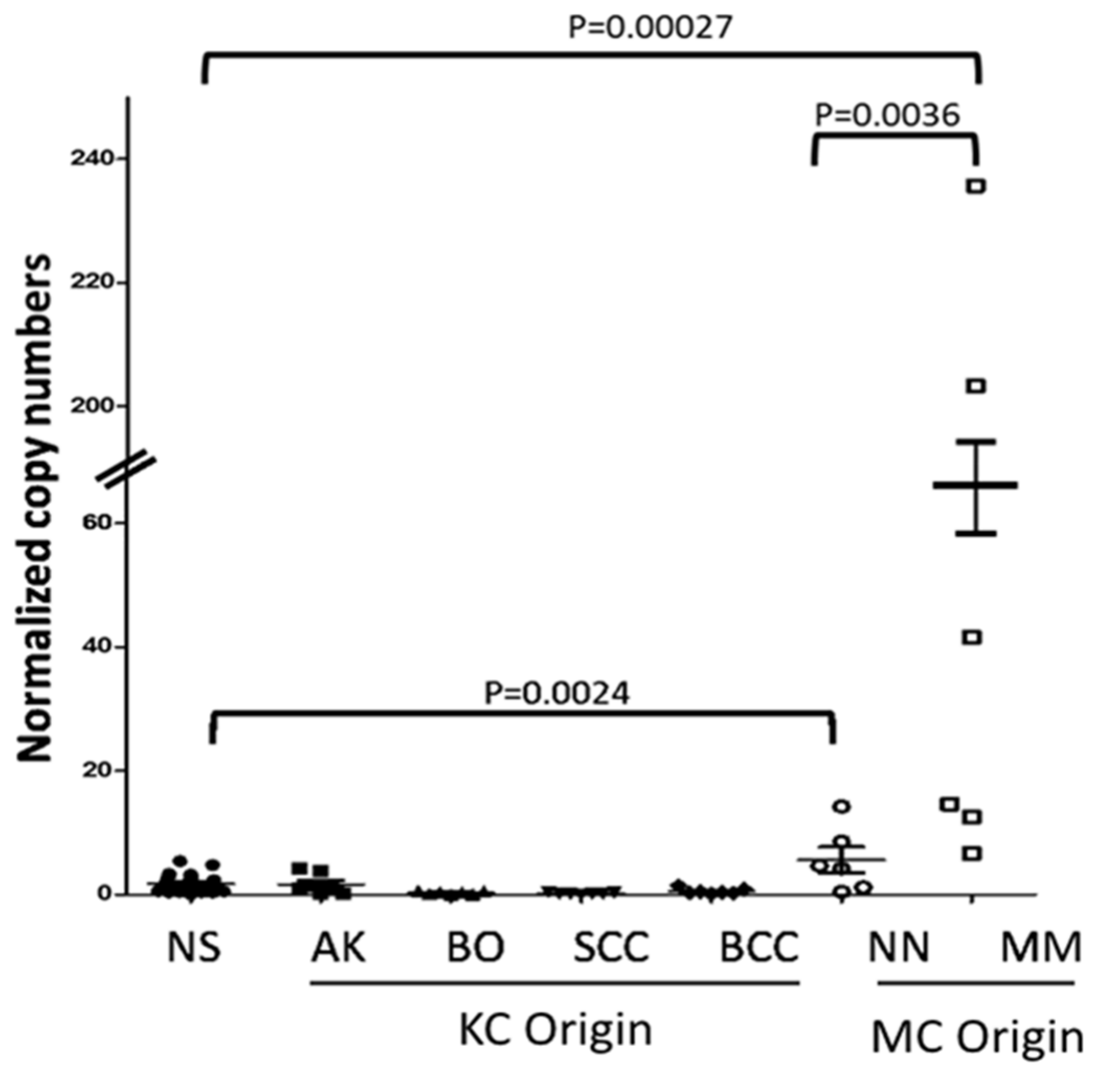
EYA1 expression in melanocytic and non-melanocytic skin tissues EYA1 expression in normal skin (NS) and skin neoplasms such as actini keratosis (AK), Bowen’ disease (BO), squamous cell carcinoma (SCC), basal cell carcinoma (BCC), (NN), and malignant melanoma (MM), were quantified by RT-PCR. The expression levels are normalized to 1000 copies of GAPDH mRNA levels. ^*^ p<0.05.

Given that UV induced DNA damages are the main determinants of malignancies arising from both keratinocytes and from melanocytes in the skin, we first examined the expression levels of EYA1 mRNA in skin biopsies of keratinocyte-derived and melanocyte-derived skin tumors using normal skin (NS) biopsies as the control. The keratinocyte derived tumors or precursors include actinic keratosis (AK), Bowen's disease (BD, or squamous cell carcinoma *in situ*), invasive squamous cell carcinoma (SCC), and basal cell carcinoma (BCC). The melanocyte derived tumors include benign nevi (BN), dysplastic nevi (DN), melanoma *in situ* (MIS), primary melanoma (PM), and metastatic melanoma (MSM). As shown in Figure 1, EYA1 mRNA expression was low in all keratinocyte derived tumors, but was increased in benign melanocyte tumors (NN) (P = 0.0024). Interestingly, there was a dramatic up-regulation of EYA1 mRNA in malignantly transformed melanocytic tumors MM(P = 0.00027).

**Figure 2:**
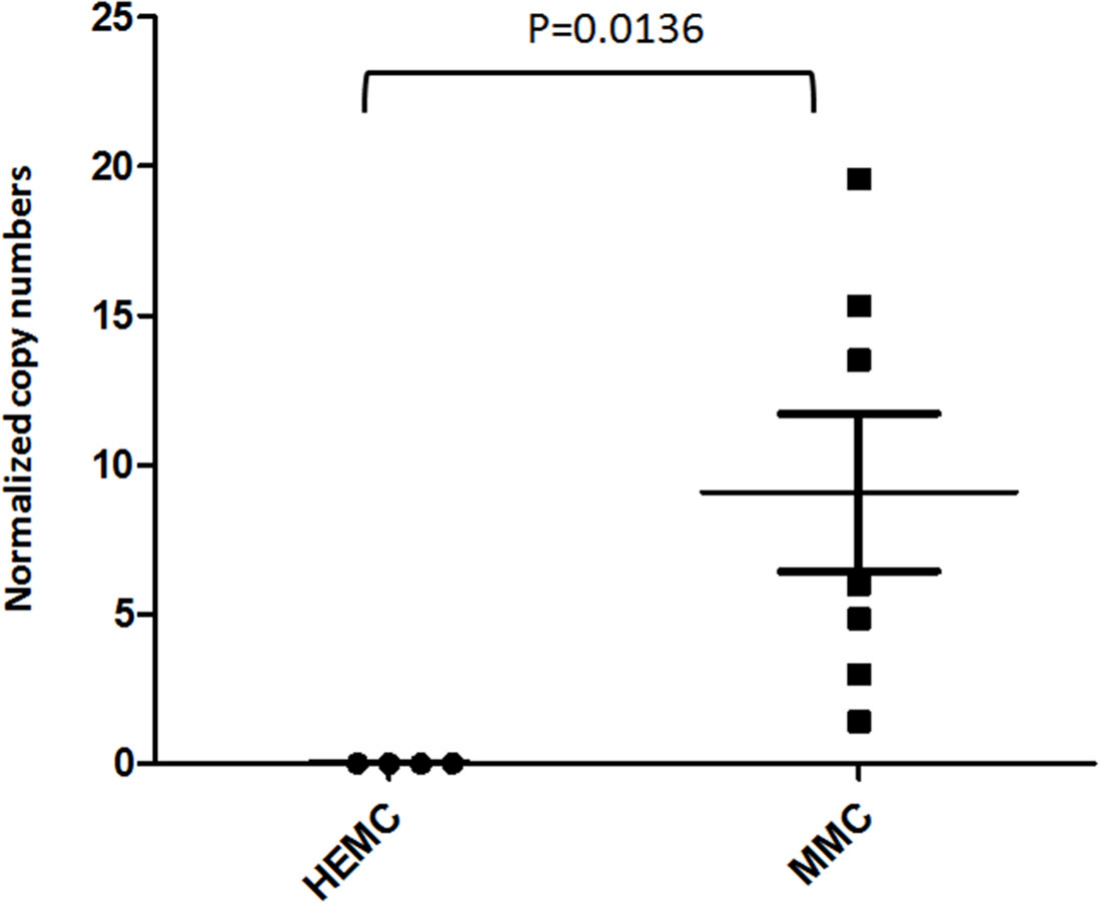
EYA1 upregulation is preserved in melanoma cell lines EYA1 messenger RNA levels in cultured cells lines were determined using quantitative RT-PCR. The levels were expressed as copies of EYA1 mRNA per 1000 copies of GAPDH. Abbreviations: HEMC: human epidermal melanocytes; MMC: malignant melanoma cells.

We next examined if the expression of EYA1 in melanoma biopsies is preserved in cultured melanoma cells by comparing the expression of EYA1 mRNA in cultured primary melanocytes (HEMC) and patient-derived melanoma cell lines (MMC). As can be seen in Figure 2, the upregulation of EYA1 is maintained in long-term cultured melanoma cells (P = 0.0136).

### EYA1 upregulation correlates with malignant transformation and increased mitosis

**Figure 3:**
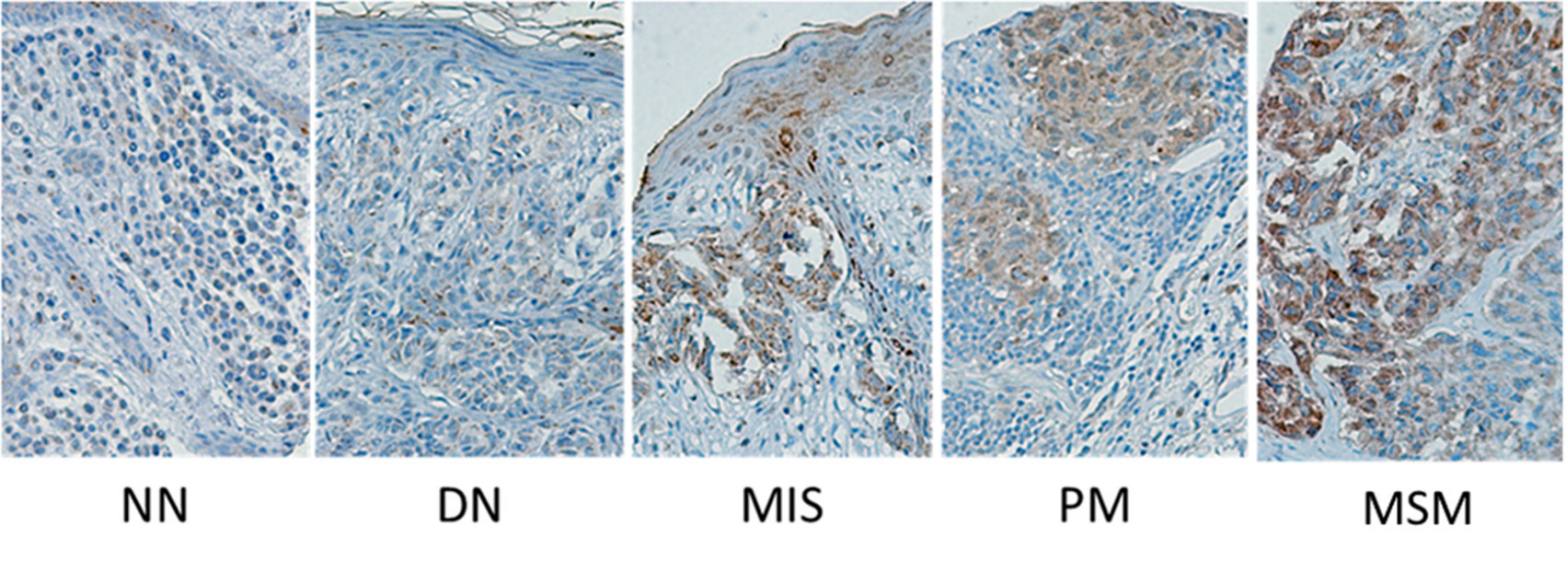
Tissue micrographs of immunohistochemistry staining of melanoma biopsies Biopsies were classified based on cancer progression: normal nevi (NN), dysplastic nevi (DN), melanoma *in situ* (MIS), primary melanoma (PM), and metastatic melanoma (MSM).

To further evaluate the significance of EYA1 expression in melanocytic tumors, we performed immunohistochemistry analysis on a spectrum of benign and melanocytic tumors (Figure 3), including BN, DN, MIS, PM and MM, using previously constructed melanoma tissue microarrays that contains 326 melanoma biopsies that have been annotated with clinical and pathological parameters of melanoma patients. The expression was low in BN and DN, but was dramatically upregulated in MIS, and remained high in PM and MM (Table 1), indicating that EYA1′s aberrant expression is initiated during the malignant transformation step of melanoma progression.

**Table 1:** Melanoma clinical pathological parameters and EYA1 staining intensities^#^

Clinical Pathological Parameters	Total number	Intensity of EYA1 Staining				*P* value (χ^2^ test)
		0	1	2	3	
**Lesion Type**						
NN	24	5(21%)	8(33%)	9(38%)	2(8%)	p<0.0001^*^
DN	38	8(21%)	12(32%)	12(32%)	6(16%)	
MIS	14	0(0%)	3(21%)	3(21%)	8(57%)	
PM	177	13(7%)	35(20%)	48(27%)	81(46%)	
MM	135	9(7%)	22(16%)	60(44%)	44(33%)	
**AJCC Stage of Melanoma**						
I	76	6(8%)	18(24%)	21(28%)	31(41%)	P=0.0680
II	101	7(7%)	17(17%)	27(27%)	50(50%)	
III	82	6(7%)	10(12%)	34(41%)	32(39%)	
IV	53	3(6%)	12(23%)	26(50%)	12(23%)	
**Age**						
≤ 60	157	10(6%)	30(19%)	51(32%)	66(42%)	p=0.8902
> 60	152	12(8%)	30(20%)	52(34%)	58(38%)	
**Sex**						
Male	184	14(8%)	39(21%)	64(35%)	67(36%)	p=0.4326
Female	125	8(6%)	21(17%)	39(31%)	57(46%)	
**Tumour Breslow Thickness (mm)**						
≤1	31	4(13%)	7(23%)	11(35%)	9(29%)	p=0.0414^*^
>1	147	9(6.1%)	28 (19%)	38(26%)	72(53%)	
**Mitosis**						
Present	112	7(6%)	20(18%)	26(23%)	59(53%)	p=0.0374^*^
Not present	65	6(9%)	16(25%)	22(34%)	21(32%)	
**Tumor-infiltrating lymphocytes**						
Not present	70	6(9%)	12(17%)	23(33%)	29(41%)	p=0.3213
Non-brisk	91	5(5%)	20(22%)	19(21%)	47(52%)	
Brisk	15	2(13%)	3(20%)	6(40%)	4(27%)	
**Ulceration**						
Present	55	3(5%)	11(20%)	14(25%)	27(49%)	p=0.8651
Not present	121	10(8%)	24(20%)	34(28%)	53(44%)	
**Regression**						
Present	10	2(20%)	2(20%)	2(20%)	4(40%)	p=0.3281
Not present	164	9(5%)	33(20%)	46(28%)	76(46%)	
**Histological Satellitosis**						
Present	8	0(0%)	3(38%)	1(13%)	4(50%)	p=0.4513
Not present	168	13(8%)	32(19%)	47(28%)	76(45%)	

^#^χ2 test was performed to evaluate the correlation between EYA1 staining and various parameters. ^*^ p<0.05 is considered to be statistically significant.

To further investigate the clinical relevance of EYA1 protein expression, we performed regression analysis between EYA1 expression intensity and patient's age, gender, AJCC staging, tumor thickness, mitotic rate, melanoma subtypes, and other clinical pathological parameters. As shown in Table 1, increased EYA1 protein expression is highly correlated with increased mitotic rate and tumor thickness. These findings suggest that the increased EYA1 expression may contribute to malignant transformation and accelerated cell division in melanoma. Furthermore, those with a higher EYA1 expression generally show a lower survival rate, although the trend is not significant (p = 0.205, χ^2^ test).

### EYA1 promotes melanoma growth by increasing DNA synthesis and cyclin D1 expression

**Figure 4:**
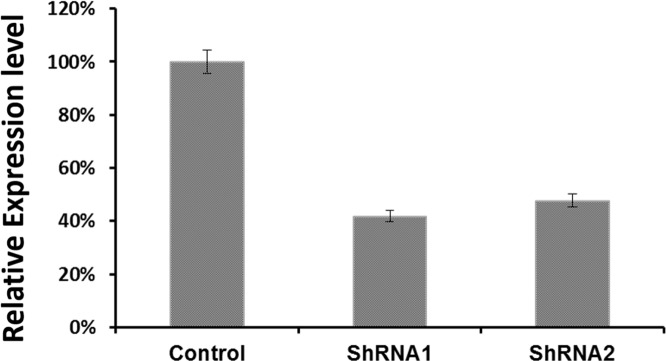
Retroviral vector mediated EYA1 knockdown in melanoma cells Retroviral vectors were used to express siRNA in a stable fashion in melanoma cells as described in the Methods section of the text. To remove variance due to clonal heterogeneity, bulk stable cells in P2 passages of the transfected cells were used to evaluate the degree of EYA1 gene knockdown, and for phenotypic assays. The mRNA levels of EYA1 is expressed as copies per 1000 copies of GAPDH. ^*^ p<0.05.

**Figure 5:**
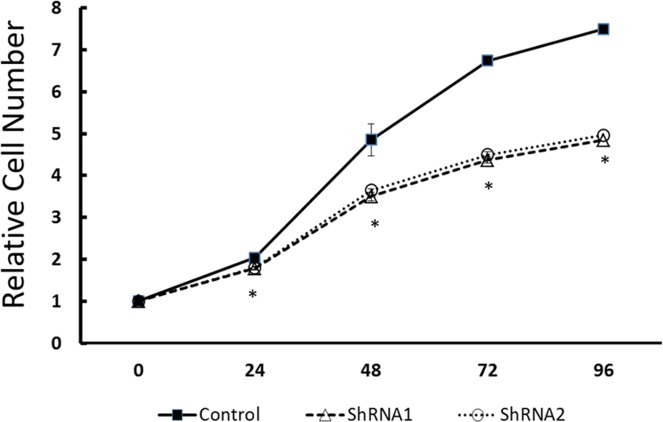
Effect of EYA1 knockdown on melanoma cell growth The control vector-containing A375 cells as well as stable cells containing vectors with ShRNA 1 or ShRNA 2 were cultured as described in the text. The cell mass was determined at specific time points by CTB method as described in the text. ^*^ p<0.05.

**Figure 6:**
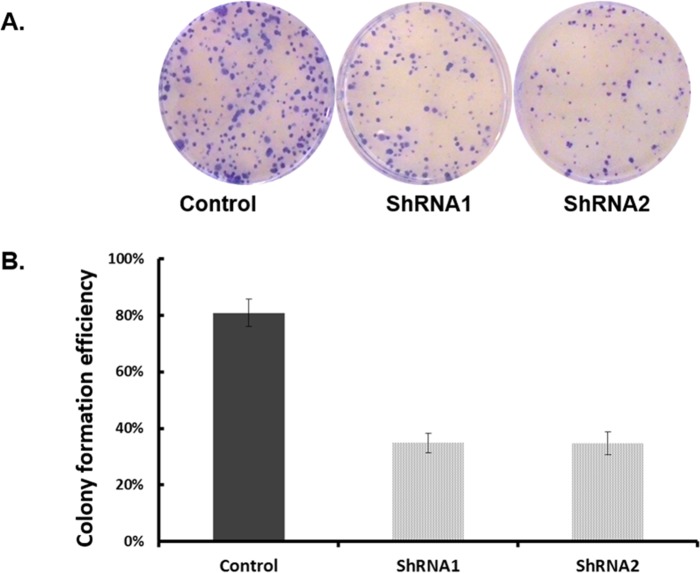
Effect of EYA1 knockdown on colony formation of melanoma cells A375 cells containing retroviral vectors (control, ShRNA1, ShRNA2) were plated on soft-agar plated in standard culture conditions as described in the text. At the end of the culture period of two weeks, the plates were stained and photographed (**Panel A**) and the number of colonies were counted (**Panel B**). ^*^p<0.05

**Figure 7:**
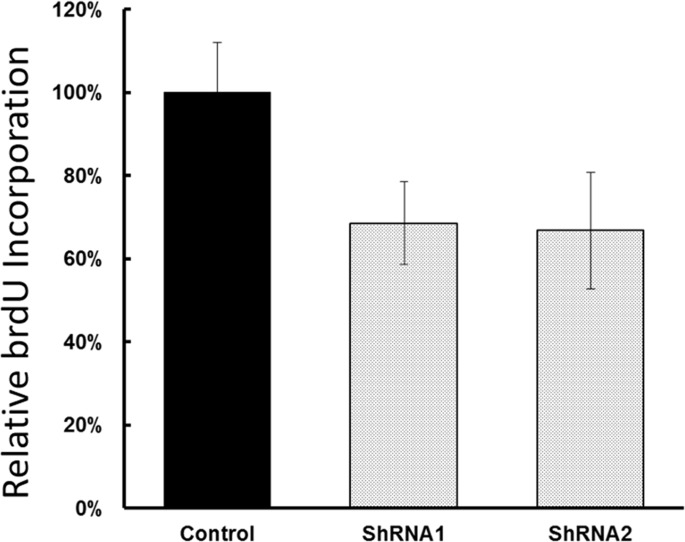
Effect of EYA1 knockdown on DNA Synthesis in melanoma cells A375 cells containing retroviral vectors (control, ShRNA1, ShRNA2) were treated with medium containing BrdU, and the amount of incorporation was determined and plotted. ^*^ p<0.05.

To examine the functional significance of EYA1 upregulation in melanoma cells, ShRNA was used to silence expression of EYA1 in cultured A375 cells. As shown in Figure 4, levels of EYA1 mRNA were significantly reduced (up to 60%) in a stable fashion by retroviral-mediated ShRNA knockdown. Melanoma cells with down-regulated EYA1 had much lower levels of cellular growth rate (Figure 5), decreased colony formation (Figure 6), and reduced DNA synthesis (Figure 7). In addition, SH4, when treated with ShRNA, also had significantly reduced proliferation rate (data not shown). However, there was no significant change in apoptosis rate, or cellular migration (data not shown). These suggest that EYA1 upregulation primarily altered rate of cellular proliferation in melanoma cells.

**Figure 8:**
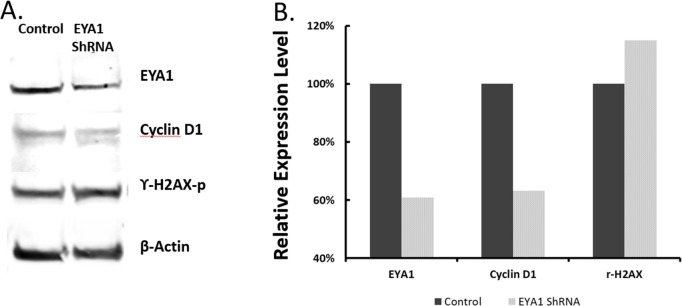
Effects of EYA1 knockdown on expression of cyclin D1 and phosphorylated γH2AX histone Immunoblots were performed using specific antibodies against cyclin D1 and phosphorylated γH2AX proteins using cell lysates prepared from stable A375 melanoma cells containing ShRNA vectors (control vector, and vectors with ShRNA1 or ShRNA2 of EYA1 gene), as described in the text. **Panel A**: Representative immunoblots using antibodies against EYA1 (Proteintech 22658-1-AP, 1:600), Cyclin D1 (Santa cruz, sc-20044, 1:100), ϒ-H2AX (Abcam, ab11174 1:1000), and β-Actin (Abcam, [AC-15] ab6276, 1:1000); **Panel B**: Expression levels were quantified using ImageJ software and normalized to β-Actin. Plotted are averages of three independent experiments. ^*^ p<0.05.

We next examined the status of the down-stream molecular changes in EYA1-silenced melanoma cells. Since EYA1 was reported to increase cyclin D1 production and decrease phosphorylated γH2AX in breast cancer cells [29], we examined if these were the case in melanoma cells. As shown in Figure 8, when EYA1 expression was reduced, there was a corresponding reduction of cyclin D1 expression. Further, EYA1 reduction in melanoma cells caused reduction of its phosphatase function as a result of increased phosphorylation of histone γH2AX (Figure 8A).

### EYA1 inhibitor benzbromarone decreases melanoma cell proliferation and enhances responsiveness to vemurafenib therapy

**Figure 9:**
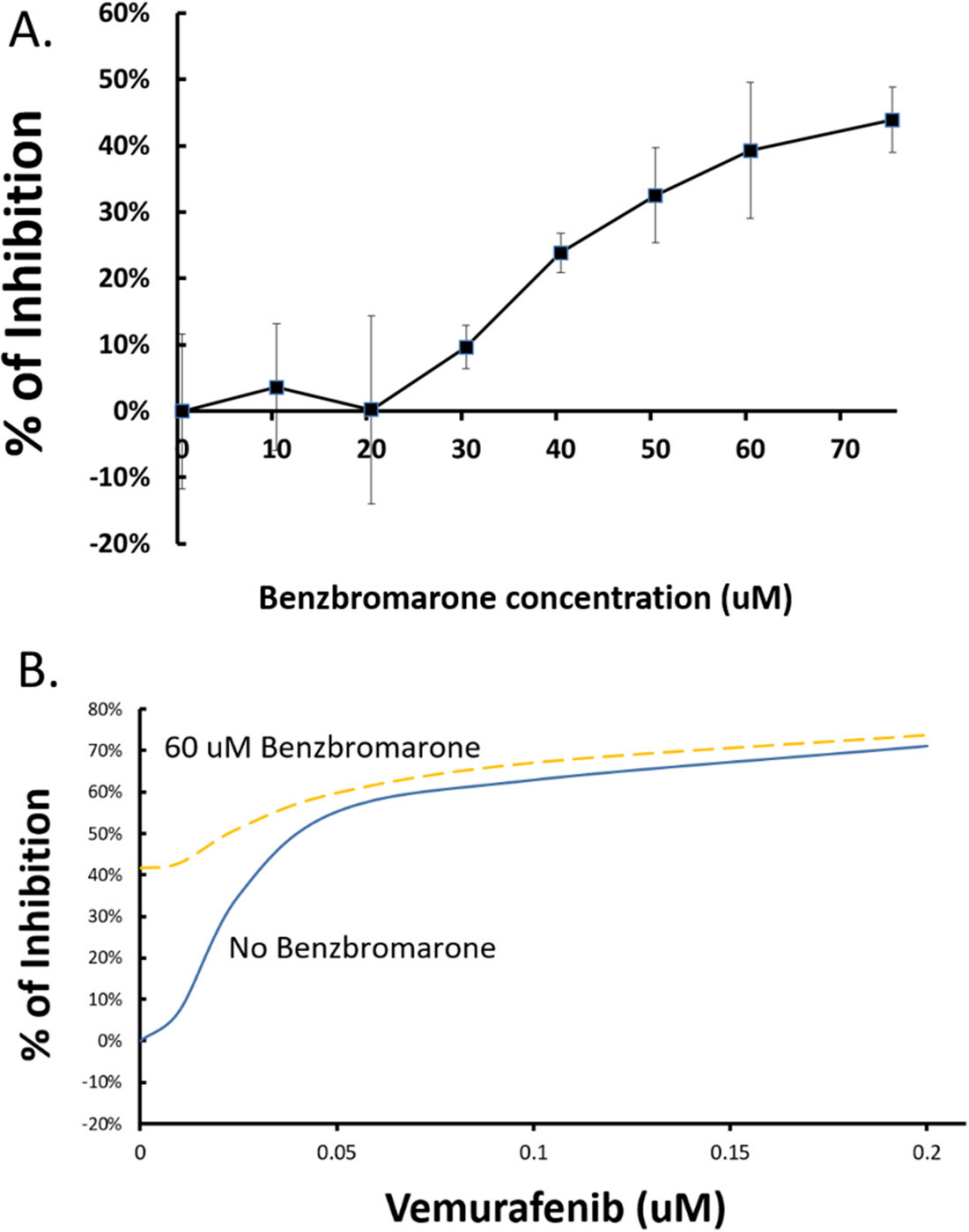
Effects of benzbromarone on A375 proliferation and sensitivity to vermurafenib **Panel A**: Various concentrations of benzbromarone in DMSO was added to the growth medium and A375 cells were grown for 48 hours and the cell density of the A375 cells were measured using CTB protocol as described in the text. The degree of growth inhibition at each concentration was plotted. ^*^ p<0.05 as compared with the control. **Panel B**: Vermurafenib was added to the growth medium of A375 cells at variable concentrations as shown, with or without 60 uM of benzbromarone. The resultant cell numbers were determined using CTB protocol, and the results plotted using the averages of three independent experiments. ^*^ p<0.05.

To further demonstrate the therapeutic implications of EYA1 in melanoma, we tested if chemical inhibitor of EYA1 has any effects on the proliferation of melanoma cells. As shown in Figure 9A, benzbromarone, which has been shown to have significant inhibitory effect on the phosphatase activity of EYA proteins [30], showed dose-dependent and significant inhibition on melanoma cell growth. Furthermore, benzbromarone increased sensitivity of melanoma cells to BRAF inhibitor vemurafenib (Figure 9B) in A375 melanoma cells (which carries BRAF V600E mutation), suggesting that EYA1 inhibitor may be a useful therapeutic choice both as a monotherapy and as an adjunct to existing targeted melanoma therapy.

## DISCUSSION

During the complex and orchestrated process of melanoma progression, numerous genes are turned on, some early, and, and others late in the process [3]. Early increased genes such as cyclin D1, primarily are involved in promotion of cellular proliferation, whereas late up-regulated genes are involved in modification of the tissue microenvironment and interaction with the host defense and immune surveillance mechanisms, such as osteopontin [13], SERPINA3 [12, 31], and CTHRC1 [23]. The results presented in this study showed that EYA1 upregulation occurred early in the malignant transformation of melanoma cells, starting as early as in melanoma *in situ* stage during radial growth phase. Consistent with this, *in vitro* functional characterization indicated that it promotes cellular proliferation and colony formation, associated with increase in DNA synthesis (BrDU incorporation) and increased cyclin D1 expression.

Given the similarity of our results and those conducted by Cook *et. al* [27], we propose a mechanism of action of EYA1 in the pathogenesis of melanoma resembling its role in the pathogenesis of breast cancer. EYA1 expression results in a tyrosine phosphatase that can dephosphorylate the pro-apoptotic histone γ2AX [27], thus promoting DNA repair and cellular proliferation.

Our results revealed that EYA1 may be a valuable target for developing novel therapies of melanoma, which, despite the recent significant therapeutic developments, still carries high mortality. Indeed, when added to cultured A375 melanoma cells, benzbromarone, an inhibitor of the phosphatase activities of EYA protein family, resulted in significant reduction of viability of the melanoma cells, and made them more susceptible to treatment with the current targeted therapy of melanoma, vemurafenib, further supporting the potential of this approach. However, benzbromarone's synergistic effect was gradually weakened when vemurafenib's concentration increases. The mechanism behind this synergistic effect is not clear; it would be an interesting topic for further investigation.

In conclusion, our study showed significant aberrant upregulation of EYA1 phosphatase early in the melanoma transformation process, and that inhibition of this enzyme either by gene silencing or by chemical agents leads to inhibition of melanoma cell proliferation. Therefore, EYA1 signaling pathway may represent an attractive target for developing melanoma therapies in the future.

## MATERIALS AND METHODS

### Clinical biopsy samples and cell lines

The experiments were performed in accordance with the Declaration of Helsinki guidelines. The study was approved by the Clinical Research Ethics Board of the University of British Columbia (Certificate H12-02653). With informed consent, biopsies were obtained and stored in RNAlater solution (Life Labs) as previously described [23, 31] [32]. Biopsy tissues were archived and stored in −20°C in RNAlater solution (Invitrogen, Canada). Human epidermal melanocytes were purchased from ScienCell (Carlsbad, USA) and cultured in melanocyte medium (2201, ScienCell, Carlsbad, USA). Melanoma cell lines A375, RPMI 7951, SH4, WM-115, SK-MEL-1, SK-MEL-3, SK-MEL-24 were purchased from ATCC (Manassas, USA). Cells were cultured in growth medium as Dulbecco's modified Eagle's medium (DMEM) (Hyclone, Logan, USA) supplemented with 10% fetal bovine serum (Hyclone, Logan, USA) and 1X Antibiotic-Antimycotic (15240062, Gibco, Burlington, Canada) at 37°C in 5% CO2 humidified atmosphere.

### RNA preparation and quantitative PCR

RNA from biopsy samples were extracted in Qiazol solution using homogenizer followed by RNeasy kit (Qiagen, Toronto, Canada). RNA prepared from cultured cells was using RNeasy kit. RNA was quantified using absorbance 260 nm and evaluated the purity by ratio of 260/280 (Epoch, Take3 Micro-Volume Plates, BioTek, Winooski, USA). 1ug of RNA was used for reverse transcription reaction (SuperScript® VILO cDNA Synthesis Kit, Life Technology, Burlington, Canada). 1/200 of synthesized cDNA was setup for real-time PCR using SYBR selection master mix (Life Technology, Burlington, Canada) on a StepOne Plus real-time PCR system (Life Technology, Burlington, ON). The following primers are used to evaluate the expression level normalized with internal *GAPDH* control. (Primers for EYA1: forward-GGACAGGACCTAAGCACATA;reverse-GTACACCAGTTGCCAAACAT; Primers for GAPDH: forward-AAGATCATCAGCAATGCCTCC; reverse- TGGACTGTGGTCATGAGTCCTT).

### Immunohistochemistry (IHC) staining of tissue microarrays

The selection of melanoma tissue blocks and construction of tumor tissue microarrays have been described previously [6, 7, 12, 33–37]. The formalin-fixed, paraffin embedded archival biopsies of benign melanocytic nevi, melanoma *in situ*, invasive primary melanoma and metastatic melanoma were obtained from the 1990 to 2009 archives from the Department of Pathology at Vancouver General Hospital, Vancouver, Canada. A total of 411 biopsies were available to evaluation by immunohistochemistry, including 24 normal nevi, 38 dysplastic nevi, 14 melanoma *in situ*, 177 primary melanoma, and 135 metastatic melanoma samples. The clinicopathological data was available for all melanoma cases.

EYA1 expression was analyzed by immunohistochemistry on paraffin-embedded tissue microarrays as described previously [6, 7, 12, 33–37]. Briefly, de-paraffinized 4μm tissue sections were treated 30 minutes in 0.1M sodium citrate (PH6.0) at 95°C for antigen retrieval. Slides were then treated with 3% hydrogen peroxide for 30 minutes to block endogenous peroxidase activities. After blocking with protein block serum free solution (X0909, DAKO, Carpinteria, USA), anti-EYA1 rabbit polyclonal antibody (22658-1-AP, Proteintech, Rosemont, USA) was in antibody diluent (S0809, DAKO, Carpinteria, USA) as 0.25 ug/ml and applied to sections by incubating overnight at 4°C. Normal rabbit serum with same concentration was used as a negative control. 4μg/ml anti-Melan-A clone A103 mouse monoclonal antibody (M7196, Dako, Carpinteria, USA) was used on adjacent sections to locate melanocyte-derived cells. EnVision + Dual Link System –HRP (K4063, Dako, Carpinteria, USA) was applied to the sections followed by DAB Substrate-Chromogen System (K3468, Dako, Carpinteria, USA) and hematoxylin for positive and nuclei staining, respectively.

### Statistical analysis

Statistical analysis was performed with GraphPad Prism 5 software (GraphPad Software, San Diego, CA). Differences in EYA1 staining in the various stages of melanoma were evaluated using chi-squared (χ2) analysis. The effects on cell apoptosis, proliferation, migration, and matrix invasion of cultured melanoma cells were evaluated using Student's t test. The statistical significance level was set at p<0.05. Survival analysis was performed using SPSS software. Kaplan-Meier tests were used to determine significance of EYA1 intensity on survival of patients. Cox regression was used to determine independence of EYA1 as a risk factor for melanoma patients.

### Quantification of EYA1 staining intensity and statistic analysis

A previously described [6, 7, 12, 33–37] a 4-point scoring system was used to determine intensity of EYA1 staining. Scoring was performed by three independent scorers, including a dermatopathologist, without access to clinico-pathological information of the sections. Discrepancies among the scorers were resolved by obtaining a consensus score, whereby the group evaluated the sections simultaneously using scanned microscope images. In the cases with a discrepancy between duplicated cores, the higher score from the two tissue cores was taken as the final score.

### ShRNA knockdown

Two EYA1 ShRNA vectors (TRCN0000083446 and TRCN0000083443, Sigma-Aldrich, Oakville, Canada) were used for lenti-virus packaging and knockdown in cultured melanoma cell lines. None-mammalian ShRNA control plasmid (SHC002, Sigma-Aldrich, Oakville, Canada) was used as a control vector. After viral transduction, cells were cultured in growth medium containing 2 ug/ml puromycin (A1113803, Life Technology, Burlington, Canada) for selection. Cells were expanded for 2 passages and all experiments were conducted from same passage of cells.

### Viability and proliferation assay

Cells were trypsinized and seeded in triplicates on a 96 well plate at 1500 cells/well. After cultured for 4 hours (time 0), 24 hours, 48 hours and 72 hours in growth medium with 2ug/ml puromycin, cell viability was assessed using CellTiter-Blue cell viability kit (G8080, Promega, Madison, USA). Fluorescent signal generated from viable cells were measured at 560Ex/590Em using plate-reading fluorometer (Glomax, Promega, Madison, USA) after incubating for 8 hours.

Proliferation was assessed using BrdU Cell Proliferation Assay Kit (6813, Cell Signaling Technology, Whitby, Canada) following manufactory instruction. In brief, cells were seeded in triplicates with 10,000/well and cultured in growth medium containing 1X Brdu and 2ug/ml puromycin for 15 hours. Cells were then fixed and use the detection system from the kit to measure the Brdu incorporation efficiency. Absorbance signal at 450nm was measured using micro-plate reader (Epoch, BioTek, Winooski, USA).

### Chemical treatments of melanoma cells

Melanoma Cells were seeded at a density of 4000 cells/well in a 96 well plate with growth medium containing various concentration of benzbromarone (B5774, Sigma-Aldrich, Oakville, Canada) (range 0-100uM), vemurafenib (PLX4032)(CT-P4032, Chemietek, Indianapolis, USA) (range 0-0.2uM) or both. Viability of treated cells was evaluated using CellTiter 96® Aqueous One Solution MTS based proliferation assay (G3580, Promega, Madison, USA) on time 0, 48 hours and 96 hours after drug treatment. OD absorption at 490nm after 4 hours incubation with CellTiter 96 MTS solution was read using micro-plate reader. Inhibition of cell viability were calculated by comparing the background subtracted signals from DMSO only (0 uM) treated cells with the drug treated cells. Triplicate wells are seeded for each condition and the experiments were repeated 3 times. ^*^ shows significant on T-test (P<0.05).

### Colony formation assay

Cells are seeded at 700/well, 350/well and 175/wells in a 6-well plate setup with growth medium containing 2 ug/ml puromycin. After 9 days of incubation in 37 degree with 5% CO2 with changes of growth medium containing 2ug/ml puromycin every 3 days, cells were fixed with 10% formalin for 30 minutes at room temperature followed by staining with 0.01% crystal violet for 30 minutes at room temperature. Photographs of staining plates were assessed using ImageJ software to measure the colonization efficiency.

### Immunoblot analysis

Protein was extracted from cells by adding RIPA lysis buffer containing protease inhibitor cocktail (cOmplete Tablets, 04693124001 Roche, Oakville, Canada) and 4 pulse of 5 seconds sonication. Protein was quantified using BCA Protein Assay Kit (23225, Pierce, Burlington, Canada). 30ug of total protein was loaded and separated by electrophoresis on 4-12% gradient polyacrylamide gel. PVDF membrane (Immuno-blot PVDF, Bio-rad, Hercules, USA) was used for transferring blot. Membrane containing protein was incubated with 5% BSA in TBS for 1 hour at room temperature for blocking followed by incubating with 0.5ug/ml anti-EYA1 rabbit polyclonal antibody in 0.5%BSA-TBST for overnight at 4 degree. Membrane was washed 10 minutes in TBST (0.05% Tween-20) for 3 times, then incubated in 1:30,000 diluted goat anti rabbit IRDye 800 CW secondary antibody (925-68070, Li-Cor, Lincoln, USA) for 1 hour at room temperature. After washing with TBST 10 minutes 3 times, membrane was scanned on Odyssey® CLx Imaging system (Li-Cor, Li-Cor, Lincoln, USA) for visualization of the signals.

## Abbreviations

AK: actinic keratosis
BD: Bowen's disease
BCC: basal cell carcinoma
BN: benign nevi
DN: dysplastic nevi
EYA1: “eyes absent” gene
HEMC: cultured primary melanocytes
PM: primary melanoma
MMC: patient-derived melanoma cell lines
MSM: metastatic melanoma
NN: benign melanocyte tumors
NS: normal skin
SCC: squamous cell carcinoma
